# Mindfulness-based stress reduction (MBSR) effects on the worries of women with poly cystic ovary syndrome (PCOS)

**DOI:** 10.1186/s12888-023-04671-6

**Published:** 2023-03-21

**Authors:** Zahra Salajegheh, Atefeh Ahmadi, Hadis Shahrahmani, Yunes Jahani, Katayoun Alidousti, Fatemeh Nasiri Amiri, Zohreh Salari

**Affiliations:** 1 Nursing Research Center, School of Nursing and Midwifery, Kerman University of Medical Sciences, Kerman, Iran; 2 Modeling in Health Research Center, Institute for Futures Studies in Health, Kerman University of Medical Sciences, Kerman, Iran; 3 Infertility and Health Reproductive Research Center, Health Research Institute, Babol University of Medical Sciences, Babol, Iran; 4 Obstetrics and Gynecology Center, Afzalipour School of Medicine, Kerman University of Medical Sciences, Kerman, Iran

**Keywords:** Polycystic ovary syndrome, Mindfulness, Mindfulness stress reduction, Concern, Quality of life

## Abstract

**Introduction:**

Polycystic ovary syndrome is one of the women’s most common endocrine disorders that can cause anxiety, psychological distress, and reduced quality of life. Therefore, the present study aimed to determine the effect of mindfulness-based stress reduction counseling on the worries of women with polycystic ovary syndrome.

**Materials and methods:**

This quasi-experimental was implemented on 60 women with polycystic ovary syndrome, referring to health centers in Kerman, Iran, from April to September 2021. In the intervention group, MBSR was conducted in eight 90-minute sessions twice a week. A researcher-made questionnaire with 34 questions (with six domains including worries related to mental complications, interpersonal problems, non-pregnancy physical complications, pregnancy complications, sexual complications, and religious issues) on the worries of women with polycystic ovary syndrome was completed by the participants in two intervention and control groups as pre-and post-test and one month later. 22 SPSS statistical software was used for analysis.

**Results:**

The mean score of worries in the intervention group (48.18 ± 5.18) compared to the control group (75.73 ± 8.08) was significantly reduced in total and all six domains immediately after the intervention (P < 0.0001). One month later also, the total mean score of worries and subtitles decreased significantly (P < 0.0001) in the intervention group (38.27 ± 3.58) in comparison with the control group (76.13 ± 7.52).

**Conclusion:**

Results showed that the method of reducing stress based on mindfulness had caused a significant reduction in worries in the intervention group. Therefore, this method can be used to improve the mental health of this group of patients in health centers.

## Introduction

Polycystic ovary syndrome (PCOS) is one of the most common endocrine disorders in women of reproductive age, with clinical consequences such as reproductive, metabolic, psychological, and some cancers. The disease affects estimated between 4% and 20% of women worldwide [1]. The prevalence of this complication in Iran is reported to be 14.6% [2].

Physical issues such as infertility and menstrual irregularities appear to significantly reduce the quality of life and mental health of patients with this syndrome. Nevertheless, other things, such as changes in a person’s appearance, predominantly obesity, hair loss, acne, sexual behavior, anxiety, and depression, can significantly reduce the physical and mental quality of life [3, 4]. Due to their gender role, poor body image owing to obesity, acne, and increased hair growth severely leads to a decrease in the quality of life of these women. It has adverse effects on their mental state and psychological health. Hence, they need exceptional support from healthcare systems in different dimensions [5, 6].

One of the most important clinical consequences of PCOS in women is having many physical and psychological worries. Worries as a cognitive process predict risk and threat, including recurring thoughts and images, anxious topics, potentially stressful events, and potentially catastrophic consequences [7, 8]. Treatment of PCOS Worries is divided into complementary medicine and pharmaceutical medicine. Some non-pharmacological alleviating for worry include relaxation techniques, mindfulness changes, fun activities, and coping with worries [9].

According to the mindfulness approach, all thoughts that come to mind are accepted equally so that one does not judge those thoughts and accepts all of them as one’s current thoughts [10].

According to the findings of Amiri’s study, patients with Polycystic ovarian syndrome need exceptional support from healthcare workers to reduce their anxiety, and a lifestyle change can improve the management of their disease [8]. The results of another study showed that teenage girls with polycystic ovary syndrome are exposed to several concerns that endanger their mental health. Therefore, it is necessary to provide adequate and appropriate information about the disease and its consequences and psychosocial support [11].

Given the impact of polycystic ovary syndrome on women’s mental and psychological aspects, and since the previous studies conducted in Iran were on the quality of life, anxiety, and depression of these women and not their worries and concerns, and MBSR is a new approach, so the present study was conducted to determine the effect of MBSR on the worries of women with polycystic ovary syndrome.

## Materials and methods

### Design and setting

This quasi-experimental study was implemented on 60 women with polycystic ovary syndrome, referring to healthcare centers in Kerman, Iran, from April to the end of September 2021.

Participants included women with PCOS who were referred to healthcare centers in Kerman. Out of 16 health centers, eight were located in the north of the city, and eight were located in the south. The names of the centers were written on paper, and two centers from the north and two from the south of the city were selected by lottery. Convenience sampling was done in 4 selected centers.

### Sample size and sampling

Based on previous studies and using the following formula and considering the study power of 90%, the sample size for each group was estimated to be 27 people, which increased to 30 people in each group by taking into account the 10% probability of dropping the sample size. This study can detect a difference of 2 points or more between two groups with a power of 90%. Alpha is the type 1 error, which is set at 0.05. The variance is 4 (2 to the power of 2), which is the variance of the desired variable in a similar study [12].

$$\eqalign{\mathbf{n} & =\frac{2{({\mathbf{z}}_{1-\frac{\varvec{\alpha }}{2}}+{\mathbf{z}}_{1-\varvec{\beta }})}^{2}{\varvec{\sigma }}^{2}}{{\mathbf{d}}^{2}}\cr & \mathbf{d}={\varvec{\mu }}_{1}-{\varvec{\mu }}_{2}=2\cr & \varvec{\alpha }=0.05\,\,\,\,1-\varvec{\beta }=0.90\,\,\,\,\,\,\,\,\varvec{\sigma }=2}$$

To prevent the participants from communicating with each other and contaminating the information, one center was selected from each of the north and south areas of the city as the intervention group and one center for the control group. Finally, there were two centers in the whole city for sampling the intervention group and two centers for the control group samples.

Convenience sampling was done so that every woman diagnosed with PCOS, referred to one of the selected centers, was included in the study if she had the inclusion criteria and consent. This process continued until 30 people were placed in each group. Three participants in the intervention group were excluded from the study. 2 people due to unwillingness to continue participating in mindfulness sessions, and one person due to becoming pregnant.

Inclusion criteria included: Diagnosis of PCOS based on the Rotterdam method [13], age between15 to 45 years old, literate, resident of Kerman, lack of drug and other psychotropic substance use, lack of known mental disorders, lack of hyperprolactinemia, lack of special diet, lack of Cushing’s syndrome and, Adrenal Hyperplasia.

Exclusion Criteria were adverse psychological events such as the death of a first-degree relative during the study, failure to attend two counseling sessions, reluctance to continue the study, participation in counseling sessions other than the current program and pregnancy during the study, known chronic disease like; Cardiac, pulmonary, diabetes, anemia, immunodeficiency, hemophilia, Malnutrition [14].

## Measures

For the pre-assessment phase, the Demographic Information Questionnaire included questions such as age, level of education, spouse’s education, employment status, spouse’s job, weight, age of onset of menstruation, time of onset of symptoms, and a researcher-made questionnaire on the worries of women with polycystic ovary syndrome was completed by the participants in both groups. Since each person’s concerns can be different depending on the culture and religious beliefs, the existing questionnaires were not used, and it was decided to design a specific questionnaire based on the prevailing beliefs in the society and religious beliefs and taboos of expressing sexual problems. The questionnaire on the worries of women with polycystic ovary syndrome was consisted of 34 questions with six subscales which includes worries related to mental complications (8 questions), interpersonal problems (5 questions), non-pregnancy physical complications(7 questions), pregnancy complications(6 questions), sexual complications(5 questions), and religion issues(3 questions). It was sent to 10 professors for narration, which included faculty members of the midwifery and gynecology department and a psychologist. They examined each question in terms of face and content validity. Necessary changes were made based on their comments. To evaluate the reliability, the questionnaire was completed by 30 women with PCOS. Cronbach’s alpha was 95%. The questionnaire was prepared on a 5-point Likert scale, in which “always” got a score of 5 and “never” a score of 1. The lowest score is 34, and the highest score is 170. Getting a lower score was a sign of low worry, and getting a higher score was a sign of high worry.

## Intervention

After obtaining informed written consent and giving the essential information about the study’s objectives, women with PCOS completed the measures. After selecting the eligible women with a written informed consent form, sampling was started.

MBSR was conducted in eight 90-minute sessions twice a week [15] for 30 women in the intervention group. The intervention was given by ZS, who had previously been trained in this regard, and AA was the facilitator. Attendance sessions were held in a room in one of the health centers with good ventilation, observance of social distancing, and the use of personal protective equipment such as masks. At the end of each meeting, the date and time of the next meeting were announced in coordination with the participants, and the homework related to that session was explained. Regular between-session mindfulness meditation practice is among the critical factors proposed to produce the therapeutic benefits of mindfulness-based programs [16].

It should be noted that the all next meetings were held at certain times with the agreement of the attendees. The content of counseling sessions and homework are shown in Table 1. During the intervention, the control group received routine care. In terms of ethical considerations, if desired, mindfulness training was also held for them after completing the study.

**Table 1:** Summary of sessions of counseling based on the MBSR approach for the reduction of worry in women with PCOS

session	Content	Home works.
1	Greeting and declaration of counseling rules, the definition of the concepts of mindfulness and the main variables, description of the internal and external flow of the mind, Eating raisins, home works.	mindful eating and mindfulness on one of the daily habits (such as brushing their teeth) for at least 45 min, try to be mindful for the whole the day
2	Reviewing previous home works, mindful thinking, Mindful examination of the body, and sitting meditation, home works.	continuing previous homework, sitting meditation, body scan, trying to be mindful for the whole the day
3	Reviewing previous home works, focus on being present, practice seeing and hearing consciously in three minutes, focus on five senses in five minutes, home works.	continuing previous homework, the mindful listening and looking, five senses mindful exercise, try to be mindful for the whole of the day
4	Reviewing previous home works, stress and the body’s reaction, Practicing thoughts-emotions-body senses-behavior relationships, three minutes of concentration on an unpleasant event, mindful walking, home works.	continuing previous homework, mindful breathing techniques, mindful walking, mindfulness of unpleasant events, trying to be mindful for the whole the day
5	Reviewing previous home works, effective responses to stress, Three-Minute Breathing Space (3MBS), meditation in daily life, home works.	continuing previous homework, mindful breathing techniques, trying to be mindful for the whole the day
6	Reviewing previous home works, conscious mind interactions, taking care of yourself, practicing speaking and listening consciously, practicing consecutive thoughts in an hour, getting feedback from participants from practicing, and presenting homework Homemade.	continuing previous homework, mindful communication, thought door technique, trying to be mindful for the whole the day
7	Reviewing previous home works, being more careful, Mindful Yoga, making the unpleasant event enjoyable, providing homework	continuing previous homework, four-dimensional meditation, trying to be mindful for the whole the day
8	Reviewing previous home works, mountain meditation, summarization of all sessions, and homework ^[12]^.	continuing previous homework, try to be mindful for the whole the day

### Data analysis

Statistical analysis was performed on 30 people from the control group and 27 from the intervention group. The descriptive statistics were reported as frequency, percent, and mean(SD). To analyze the data, independent t-test, Chi-square test, Fisher’s exact, Mann-Whitney, and ANCOVA were used. ANCOVA was used to compare the intervention and control groups immediately after the intervention. The significance level was 0.05. SPSS version 22 of IBM company was used for analysis.

### Ethical considerations

This manuscript was derived from a master counseling in midwifery thesis (project code No. 97,001,078) and was approved by the Ethics Committee of Kerman University of Medical Sciences, Iran (the code of ethics No. Kmu.ac.ir.1398.163). Written informed consent was obtained to enter the study, and participants could easily withdraw from the study whenever they were willing. At the request of the ethics committee, the study was conducted following the Declaration of Helsinki and Ethics Publication on Committee (COPE). Unique codes were used for each participant to ensure information confidentiality.

## Results

The mean age of the samples in the intervention group was 32.25 ± 4.67 years, and the control group was 31.4 ± 4.37 years. The mean weight of the samples in the intervention group was 70.92 ± 7.75 kg and in the control group was 73.83 ± 8.79 kg. The mean age of onset of the first symptoms in the intervention group was 20.59 ± 1.69 years, and the control group was 20.8 ± 2.32 years.

The mean number of children in the intervention group was 1.4 ± 0.5, and the control group was 1.06 ± 0.98. There was no statistically significant difference between the two groups in terms of quantitative variables except the age of onset of menstruation. [Tables 2 and 3].

**Table 2:** Comparing the distribution of quantitative variables between of two intervention and control group

Group Variable	Control		Intervention	Independent T-test	P value
	Mean ± SD				
Age	31.4 ± 4.37	32.25 ± 4.67		-0.71	0.471
Weight	73.83 ± 8.79	70.92 ± 7.75		1.38	0.191
Age of Onset of Menstruation	14 ± 0.74	13.25 ± 0.59		4.12	0.001
Age of Onset of Symptoms	20.8 ± 2.32	20.59 ± 1.69		0.38	0.700
Number of Children	1.06 ± 0.98	1.40 ± 0.50		-1.62	0.011

**Table 3:** Comparing the distribution of qualitative variables between of two intervention and control group

Group Variable		Control	Intervention	${X}^{2}$	P value
		N(%)	N(%)		
Education Level	Associate Degree	8(26.7)	8(29.6)	2.75	0. 252
	Bachelor	12(40)	15(55.6)		
	Masters and PhDs	10(33.3)	4(14.8)		
Spouse Education Level	Diploma	4(13.3)	4(14.8)	*	0.133
	Associate Degree	4(13.3)	1(3.7)		
	Bachelor	13(43.3)	19(70.4)		
	Masters and PhDs	9(30)	3(11.1)		
Job	Housewife	17(56.7)	12(44.4)	*	0.345
	Employee	10(33.3)	14(51.9)		
	Freelance	3(10)	1(3.7)		
Husbands job	Employee	17(56.7)	21(77.8)	2.85	0.091
	Freelance	13(43.3)	6(22.2)		
Family history of Polycystic Ovary	Yes	5(16.7)	0	*	0.053
	No	25(83.3)	27(100)		

*Fisher Exact Test

The mean score of pre-intervention worry was 76.21 ± 8.18 in the intervention and 75.89 ± 7.77 in the control group [P = 0.231]. Immediately after the intervention, the mean score of the worry was 48.18 ± 5.18 in the intervention and 75.73 ± 8.08 in the control group [P < 0.001]. One month after the intervention, the mean score of worry was 38.27 ± 3.58 in the intervention and 76.13 ± 7.52 in the control group [P < 0.001] [Table 4].

**Table 4:** Comparison of the mean score of worry before, after, and one month after counseling in both groups

Time	Variable Group	mental complications	Interpersonal problems	non-pregnancy physical complications	pregnancy complications	sexual complications	religion issues	Total
		Mean ± SD						
Pre-test	Intervention	17.93 ± 8.32	11.2 ± 7.59	15.69 ± 8.01	13.44 ± 11.24	11.2 ± 9.71	6.72 ± 4.21	76.21 ± 8.18
	Control	17.85 ± 8.29	11.16 ± 7.28	15.61 ± 8.43	13.39 ± 10.96	11.16 ± 7.28	6.69 ± 4.41	75.89 ± 7.77
P-value		0. 189	0. 321	0.197	0.224	0.321	0.608	0.231*
Post-test	Intervention	11.33 ± 7.21	7.08 ± 4.14	9.91 ± 6.12	8.5 ± 6.25	7.08 ± 4.14	4.25 ± 3.22	48.18 ± 5.18
	Control	17.81 ± 8.26	11.13 ± 7.15	15.59 ± 8.39	13.36 ± 9.87	11.13 ± 7.16	11.13 ± 7.15	75.73 ± 8.08
P-value		< 0.001**	< 0.001**	< 0.001**	< 0.001**	< 0.001**	< 0.001**	< 0.001**
One month later	Intervention	9 ± 5.02	5.62 ± 3.31	7.87 ± 4.56	6.75 ± 4.11	5.62 ± 3.31	3.37 ± 1.18	38.27 ± 3.58
	Control	17.91 ± 8.53	11.19 ± 7.13	15.67 ± 8.36	13.43 ± 9.48	11.19 ± 7.13	6.71 ± 4.51	76.13 ± 7.52
P-value		< 0.001**	< 0.001**	< 0.001**	< 0.001**	< 0.001**	< 0.001**	< 0.001**

* Mann-Whitney ** ANCOVA

Therefore, it is clear that during the study in the intervention group, the worries were significantly reduced. There was a statistically significant difference between the two groups regarding worries during the study. In other words, the mean score of worries was significantly lower in the intervention group than in the control group immediately after and one month after the intervention.

All subscales of the worries of women with PCOS were compared between two groups. The decrease in all dimensions scores immediately after and one month after the counseling in the intervention group was statistically significant [p < 0.001] [Table 4].

**Fig. 1:**
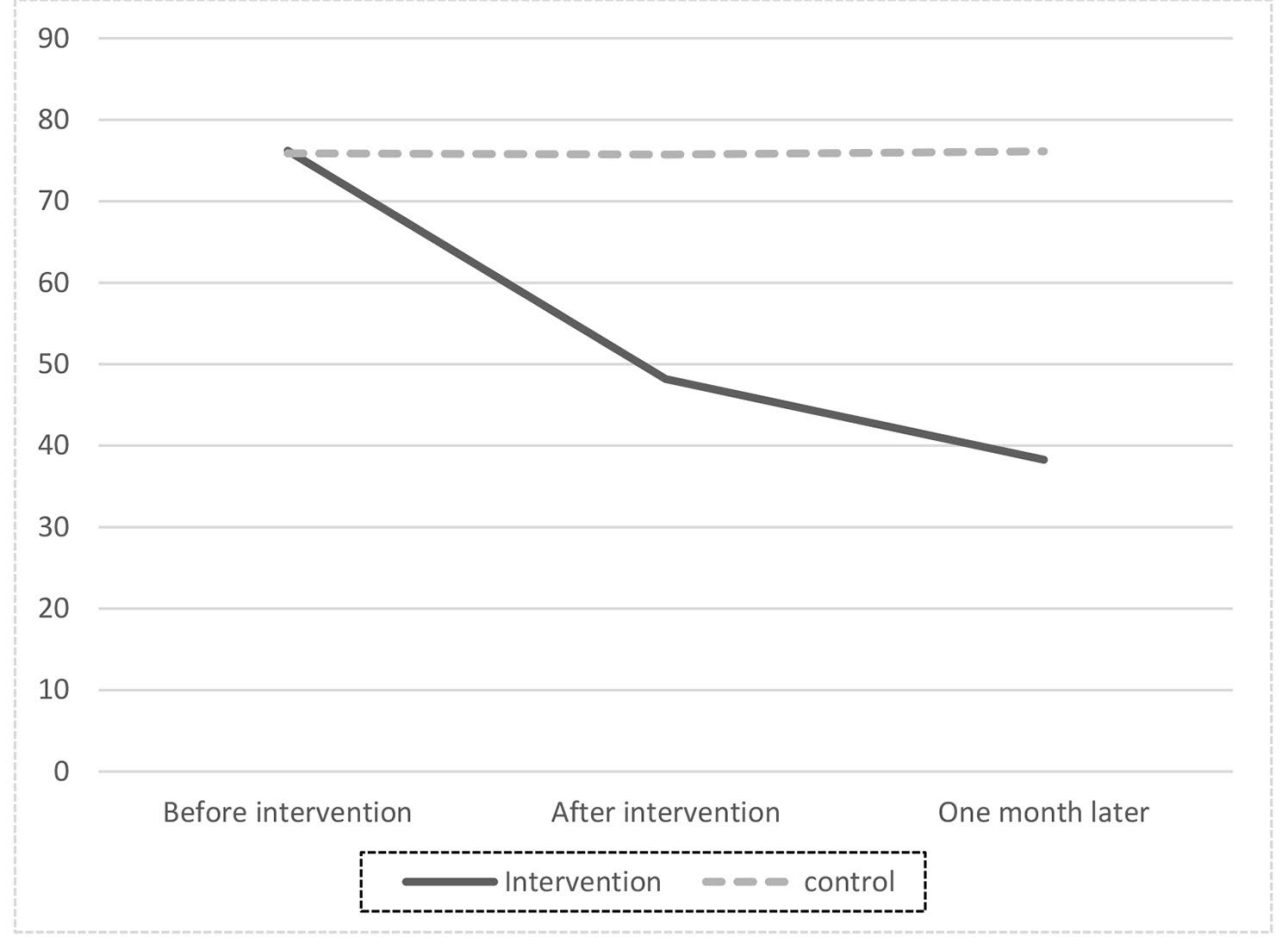
Mean of worry in both group in three different times

Repeated Measure ANCOVA Shows the difference in the level of worry during the time [Table 5]. Figure 1 also declares it better.

**Table 5:** Comparing the anxiety in two intervention and control groups (immediately and one month after the intervention) using Repeated Measure ANCOVA

Group Variable	Intervention	Control	F-test	P*
	Mean ± SD			
Worry immediately after the intervention	48.18 ± 5.18	75.73 ± 8.08	146.09	< 0.0001
Worry one month after intervention	38.27 ± 3.58	76.13 ± 7.52		

* ANCOVA adjusted by menstruation age and worry before the study

## Discussion

The present study’s findings showed that, in general, the mean score of worry in women with PCOS in the intervention group has decreased. This means that MBSR has been effective in reducing worry scores.

Since the worry includes different areas, multiple studies have been conducted, each of which has examined one branch. The worry about physical complications related to PCOS was one of the subtitles of concern, and the findings showed that MBSR was able to reduce its mean score. Our findings are in line with Lara’s study, which found that physical resistance training programs significantly reduced pain and total depression and anxiety scores in PCOS women [17].

Stress and anxiety are commonly seen in women with PCOS; according to the results, MBSR could reduce these mental problems in the samples, which is aligned with Stefanaki et al. (2015), who found in their 8-week mindfulness stress management program that there were statistically significant reductions in stress, depressive and anxiety symptoms and increase in quality of life of women with PCOS after intervention [18].

Also, the available evidence shows that exercise improves the quality of life and reduces the physical discomfort of PCOS. Nevertheless, it is practical to some extent for the symptoms of depression and anxiety of women with polycystic ovary syndrome [19]. Patel (2020) also showed that the women with PCOS who completed the mindful yoga intervention had significantly lower free testosterone and dehydroepiandrosterone levels. Additionally, improvements were seen in measures of anxiety and depression [20]. However, a meta-analysis reveals that CBT has apparent advantages in alleviating PCOS patients’ anxiety symptoms and improving their quality of life in aspects of hirsutism but No differences in depression [21].

Cognitive and behavioral treatments for anxiety have been advanced by the application of mindfulness practices. The treatment principles of MBSR are compatible with standard CBT [22]. Interestingly, a study showed worry levels were eased only in the mindfulness-based intervention group in comparison with CBT one [23]. At the same time, another research presented more effectiveness of MBSR than CBT for social anxiety disorder [24]. Stress-reducing effects of MBSR are due to improvements in perseverative cognition and emotion regulation that cut across stress-related disorders [25].

Worries as a cognitive process predict risk and threat, including recurring thoughts and images, anxiety, potential stressful events, and potentially catastrophic consequences [7]. According to the results of the present study, mindfulness reduces anxiety. It may lead to reduced pessimism. This is aligned with an interventional study evaluating the effectiveness of mindfulness training in increasing optimism in diabetic pregnant women [26].

One of the issues that follows the disease of polycystic ovary syndrome is the change in the person’s appearance so that the person suffers from hair loss, acne, hair growth in parts of the face, and obesity. Communicating with others, be it with spouses, colleagues, or other members of society, is problematic due to the decrease in self-confidence and self-esteem in this group of women, and they are concerned about starting and continuing a relationship. Fortunately, we saw that MBSR could improve interpersonal problems.

Our data is in line with the findings of a clinical trial that declared that Group counseling based on acceptance and commitment therapy could decrease body image concerns and increase self-esteem [27]. According to a study of the two therapies, Mindfulness-based Cognitive Therapy (MBCT) was more effective than acceptance and commitment therapy for improving body image concerns and emotional self-regulation [28].

Worries about sexual issues may arise from a change in appearance, called sexual anxiety. Anxiety and worry about sexual function destroy the relationship between couples and deprive the parties of the necessary self-confidence [29]. In the present study, participants were worried about sexual issues. They thought the disease might cause sexual dysfunction in them or their spouse, but mindfulness intervention could reduce their worries. As Physical resistance training program in Lara’s study significantly enhanced the total score and the desire, excitement, and lubrication domains of the FSFI in PCOS women [17].

The intervention effect continued until one month after the intervention, as seen in the other studies [17, 27, 30]. A one-month follow-up is a medium-term follow-up and shows the continuity of the intervention effect. The permanence of the intervention’s effect shows the technique’s relevance and correct implementation of the technique and the precision of the intervention.

The present study, like any other study, had limitations. One of the research limitations was that single people wanted to avoid participating in the study, perhaps because married people were more familiar with the problems caused by the disease and had touched it objectively. The other limitation is that a considerable proportion of the sample was educated, which favors the outcome. Another limitation was the need for long-term follow-up to understand the impact of the intervention in different dimensions in the long term.

## Conclusion

Overall, given the theoretical underpinnings discussed and the present study’s findings, it shows that mindfulness-based stress reduction programs have an impact on reducing worries in women with polycystic ovary syndrome. Since MBSR is a non-pharmacological method, health personnel can use it to reduce patients’ problems in health centers.

## Data Availability

The datasets used during the current study are available from the corresponding author upon reasonable request.

## Abbreviations

MBSR: Mindfulness-Based Stress Reduction
PCOS: Poly Cystic Ovary Syndrome
MMA-M: Multidimensional Mortality Awareness Measure
